# Mercury as a Remedy

**Published:** 1864-04

**Authors:** Thomas Pope

**Affiliations:** Cleobury Mortimer


### MERCURY AS A REMEDY.

LETTER FROM THOMAS POPE, ESQ.

SIR,—The homœopathist and the proscriber of mercury are in a pitable state; and woe to their victims. As with the former, so is it with the latter; many of those professing their craft publicly, like those following in their wake, recreants as they are, courting popularity, secretly belie themselves.

What may we infer from the remark of a gentleman who lately, at the Harveian Society, boasted that for several years past he had never used mercury internally? If mercury in the system be always deleterious, then I should say, the sooner such a notion be exploded, the better; for I am bold to say that I look upon mercury as an internal and external agent of more value to the medical man, in his treatment of disease, than, I had well-nigh said, all the other articles of the materia medica conjoined. Give me this, and air, food, sleep, excretions, exercise, and the passions, and I think I could do my patients more good without any of the other articles, valuable as they are.

Murcury, like arsenic and other poisonous articles of the materia medica, requires the greatest caution in its use. Who would give it in cases where the crasis of the blood is in a dissolved state, or in organic disease, particularly of the liver and heart? Or who would venture on large doses without first knowing whether idiosyncrasy existed in the individual? We know there are some few who cannot tolerate it at all, and others to whom we can hardly give enough, and, between these two states, gradual steps; therefore we see the great advantage of a knowledge of the constitution, not only of a tolerance of this, but of the others. Much may be said in the praise of this mineral, and some I have said in this JOURNAL; and I believe I can safely say that I had never cause to regret its use. I will, however, conclude with its advantage in infantile syphilis. I know four grandfathers whom I treated, before they were a month old, with a grain of blue pill daily for about two months; they have healthy progenies. For about sixty years up to the present time, I have never been long without patients of this description, male or female; so that I am not in the same predicament as the Irish candidate mentioned in our JOURNAL of the 23d ult., (page 110); and but for its use, most of them would have fallen sacrifices like the thousands of others without this specific, or would have lingered and died miserably at different periods of life; and if after marriage and progeny, what horrors ensue! I am, etc., THOMAS POPE.

*Cleobury Mortimer, February 8th,* 1864.

